# Five questions to consider before conducting a stepped wedge trial

**DOI:** 10.1186/s13063-015-0841-8

**Published:** 2015-08-17

**Authors:** James R Hargreaves, Andrew J Copas, Emma Beard, David Osrin, James J Lewis, Calum Davey, Jennifer A Thompson, Gianluca Baio, Katherine L Fielding, Audrey Prost

**Affiliations:** Department of Social and Environmental Health Research, London School of Hygiene and Tropical Medicine, London, UK; MRC Clinical Trials Unit, University College London, London, UK; Department of Clinical, Educational and Health Psychology, University College London, London, UK; Department of Epidemiology and Public Health, University College London, London, UK; Institute for Global Health, University College London, London, UK; Department of Infectious Disease Epidemiology, London School of Hygiene and Tropical Medicine, London, UK; Department of Statistical Science, University College London, London, UK

**Keywords:** Methodology, Public health, Stepped wedge trials

## Abstract

Researchers should consider five questions before starting a stepped wedge trial.

*Why are you planning one?* Researchers sometimes think that stepped wedge trials are useful when there is little doubt about the benefit of the intervention being tested. However, if the primary reason for an intervention is to measure its effect, without equipoise there is no ethical justification for delaying implementation in some clusters. By contrast, if you are undertaking pragmatic research, where the primary reason for rolling out the intervention is for it to exert its benefits, and if phased implementation is inevitable, a stepped wedge trial is a valid option and provides better evidence than most non-randomized evaluations.

*What design will you use?* Two common stepped wedge designs are based on the recruitment of a closed or open cohort. In both, individuals may experience both control and intervention conditions and you should be concerned about carry-over effects. In a third, continuous-recruitment, short-exposure design, individuals are recruited as they become eligible and experience either control or intervention condition, but not both.

*How will you conduct the primary analysis?* In stepped wedge trials, control of confounding factors through secular variation is essential. ‘Vertical’ approaches preserve randomization and compare outcomes between randomized groups within periods. ‘Horizontal’ approaches compare outcomes before and after crossover to the intervention condition. Most analysis models used in practice combine both types of comparison. The appropriate analytic strategy should be considered on a case-by-case basis.

*How large will your trial be?* Standard sample size calculations for cluster randomized trials do not accommodate the specific features of stepped wedge trials. Methods exist for many stepped wedge designs, but simulation-based calculations provide the greatest flexibility. In some scenarios, such as when the intracluster correlation coefficient is moderate or high, or the cluster size is large, a stepped wedge trial may require fewer clusters than a parallel cluster trial.

*How will you report your trial?* Stepped wedge trials are currently challenging to report using CONSORT principles. Researchers should consider how to demonstrate balance achieved by randomization and how to describe trends for outcomes in both intervention and control clusters.

## Background

In stepped wedge cluster randomized trials (SWTs), clusters are randomly allocated to crossover to the intervention at different time-points and all clusters receive the intervention eventually [1, 2]. Stepped wedge trials are used in both explanatory and pragmatic research [3, 4]. In explanatory research, the intervention is primarily implemented to study its effect. Decisions about whether to roll out the intervention further are made after research is completed. By contrast, in pragmatic research, the intervention is primarily offered in order for it to exert its expected benefits; research insights are a secondary gain. In such situations, decisions about where and when the intervention is to be delivered will be influenced by practical concerns, although randomization may be feasible. In explanatory research, an SWT may be considered instead of a conventional cluster randomized trial, if resources are insufficient to offer the intervention to all intervention clusters simultaneously. In pragmatic research, phased introduction may be planned for logistical reasons. Stepped wedge trials do offer a rigorous research option when phased implementation is planned, but they also present challenges [5–8].

Recent debates about Phase II and III trials of candidate Ebola vaccines highlight some of these challenges [9]. Some researchers argued that a SWT should be used rather than a parallel trial, as this would be more appropriate to study the effectiveness of vaccines already tested for safety and immunogenicity in Phase I trials. Others argued that the safety of the vaccine should be reassessed, and that a SWT design would make it more difficult to determine effectiveness because of difficulties in accounting for time-varying confounding caused by changes in disease incidence and preventive behaviours [10].

These debates and the recent methodological literature highlight how much remains to be done on the uses, ethics, conduct and analysis of SWTs. In this commentary, we reflect on findings from articles presented in this issue of *Trials*, by considering five questions for researchers to consider if they are planning a SWT.

## Why are you planning a SWT?

Interviews with researchers suggest that the primary reasons they choose a SWT design relate to logistic or ethical considerations rather than design advantages [11]. Three scenarios may have particular resonance for researchers thinking of conducting an SWT.

Are you an explanatory researcher thinking that phased roll-out is the only practical way to implement your study? We advise caution. Phased implementation attracts researchers to SWT but brings its own challenges. It can require repeated training activities, sustained engagement with clusters in the control arm to avoid drop-out, and increasing workload for intervention teams over time as more clusters initiate the intervention [11]. Further, it may be difficult to ensure that a randomly determined roll-out is adhered to. These logistic constraints should be considered before deciding whether a SWT is the best option. Other variants of the cluster randomized trial design can accommodate phased implementation and should be considered [6].

Alternatively, are you an explanatory researcher arguing that a SWT is appropriate, whereas a parallel cluster randomized trial is not, because the potential benefit of the interventions seems clear, at least in principle, and the research question turns on efficacy or effectiveness in a certain context? If so, you may need to think again. Planning a SWT requires you to be clear about where the equipoise lies [12, 13]. It may lie in uncertainty about the effectiveness of an intervention whose efficacy has been established, or in uncertainty about potential efficacy in a setting that is substantially different from those of previous studies. However, the equipoise has to lie somewhere because without it there is no ethical justification for delaying implementation in some clusters [11].

Finally, are you a pragmatic researcher interested in the effects of an intervention that is being rolled out, but about which there remains much to learn in a real world setting, in a new context, or on outcomes for which it has not previously been considered? Are you working alongside implementers who say that a SWT is an option? We think that such situations offer the most convincing justifications for conducting a SWT. We reiterate that modified cluster randomized trial designs can also accommodate phased implementation [6], but in cases where a well-conducted SWT is undertaken, the design will usually lead to much stronger evidence than observational studies [14].

## What SWT design will you use?

Stepped wedge trials encompass a wide range of specific designs [15]. Copas *et al.* [14] outline in this series, for the first time, a comprehensive taxonomy in which SWTs are characterized on the basis of (i) when individual-level exposure to the treatment condition starts, (ii) the duration of exposure, and (iii) the approach to outcome measurement. This taxonomy incorporates two classic designs that currently appear in the literature: those based on a closed or an open cohort of participants. These designs can potentially suffer from carry-over effects when many individuals experience both control and intervention conditions. We also define a third commonly used design: the continuous-recruitment, short-exposure design is currently neglected in the methodological SWT literature, despite being the approach used in the first SWT conducted in the Gambia [2]. Copas *et al.* [14] discuss the different issues that affect the strengths and weaknesses of these designs; future methodological research is needed to flesh out these differences.

Two design decisions are specific to SWTs: the number of crossover points – times when clusters change from control to intervention conditions – at which the intervention is introduced, and the times between successive crossover points. Both decisions may be influenced by research, implementation or logistic concerns. Commonly, clusters are divided into groups, which are then randomly allocated to the time point at which the intervention is implemented, so that the number of groups equals the number of crossover points in the trial. Researchers need to consider the impact of different decisions on study power [5, 7] and overall study length [14]. Sometimes there is a lag between the time that a cluster crosses over and the time that the intervention can affect the outcome in individuals. In an open or closed cohort, SWT measures may be taken just before every crossover point. In this case. the time between successive crossover points can be chosen to be longer than the length of the lag period [7, 16], though as an alternative, incomplete SWTs can be used with shorter time between successive crossover points but omitting measurement collection during each cluster’s lag period.

## What analysis strategy will you use?

In SWTs, outcome data under intervention conditions will be, on average, collected later than control data. Ensuring that the primary measure of the intervention effect is unconfounded by secular change in the outcome variable is, therefore, a key challenge. A range of approaches are available [7, 16–18]. Conceptually, these approaches can be thought of in two ways. Vertical approaches compare outcomes between clusters randomly assigned to either the intervention or the control condition within the time between successive crossover points. Horizontal approaches compare outcomes before and after crossing over from the control to the intervention condition [7]. In practice, most SWTs are analyzed with cluster-random effects models and adjusted for time, thereby incorporating information from both vertical and horizontal comparisons in the intervention effect [16]. An analysis conditional on time should be most robust to secular trends, but appropriate models are not easily available for all types of outcome or SWT design. Time-varying confounding can create a situation in which intervention effect estimates from horizontal and vertical approaches differ [19]. For this reason, in this collection Davey *et al.* suggest that randomized, vertical intervention effect estimates within appropriate periods should be presented and compared with the overall intervention effect from the model [16]. There remains a need for future research on vertical approaches to the analysis of SWTs, and guidance on the conditions under which caution should be taken in interpreting mixed vertical and horizontal analyses.

Finally, many SWTs include in their primary analysis data collected before or after all clusters have crossed over to the intervention condition [15]. Sometimes these data are collected from much longer periods than the time between crossover points during the trial. However, without clusters in both conditions, it is difficult to untangle the secular trends from the intervention effect using such data. These data can indirectly provide some information on the intervention effect through assumptions made concerning secular trends and the correlation of data within clusters over time, but these assumptions might become less realistic as greater periods before or after roll-out are incorporated and bias could arise in analysis. We recommend that primary analyses be based mainly on data from those exposed to the intervention or control while clusters are in both conditions, supplemented, if available, only by data from immediately before or after the roll-out period [14].

## How big should your trial be?

Standard sample size calculations for individually and cluster randomized trials fail to accommodate the specific features of SWTs. Calculations for SWTs using a design effect or other method have been published and are suitable for some SWTs [7, 20], and a Stata routine is available for some designs [21]. Baio *et al.* [22] provide in this series examples of simulation-based calculations. Though potentially more complex to implement than current methods, these provide the greatest flexibility to accommodate the full range of SWT designs and analysis models.

In some situations, for example when the intracluster correlation coefficient is moderate or high, or the cluster size is large, SWTs analyzed using mixed models, such as those already described, provide more power than parallel cluster randomized trials with the same number of clusters and cluster size [5, 22, 23].

## How will you report the design and profile of your trial?

There are currently no CONSORT guidelines for reporting SWTs, though work is underway to produce them. Copas *et al.* [14] provide a diagram outlining key design dimensions that should be reported for all SWTs. Davey *et al.* [16] identify how trial results are reported in recent SWTs, noting limitations and substantial heterogeneity in current practice. Researchers should consider how they will assess and report balance between control and intervention conditions, since all clusters experience both conditions. Some, but not all, of the papers we reviewed attempted to formally assess balance between randomized groups [24–29]. We also recommend that SWT reports should describe trends in outcomes for both the intervention and control clusters over the study period. Again, some, but not all, of the trials we reviewed provide examples of such reporting [24–26].

## Conclusion

An ethically sound, well-designed and conducted SWT with appropriate analysis can provide strong evidence of the effects of an intervention. Such evidence should be considered of higher quality than that arising from non-randomized studies. The potential for SWTs to be deployed in pragmatic public health evaluation and to increase the quality and volume of evidence available to guide public health decisions means that their appropriate use should be encouraged.

## Abbreviations

CONSORT: Consolidated Standards of Reporting Trials
SWT: stepped wedge trial
